# 4-[(*E*)-(4-Hy­droxy­benzyl­idene)amino]-3-methyl-1*H*-1,2,4-triazole-5(4*H*)-thione

**DOI:** 10.1107/S1600536814012215

**Published:** 2014-05-31

**Authors:** Balladka K. Sarojini, Padmanabha S. Manjula, B. Narayana, Jerry P. Jasinski

**Affiliations:** a Department of Studies in Chemistry, Industrial Chemistry Division, Mangalore University, Mangalagangotri 574 199, D.K., Mangalore, India; b Department of Chemistry, P. A. College of Engineering, Nadupadavu 574 153, D.K., Mangalore, India; c Department of Chemistry, Mangalore University, Mangalagangotri 574 199, D.K., India; d Department of Chemistry, Keene State College, 229 Main Street, Keene, NH 03435-2001, USA

## Abstract

The title compound, C_10_H_10_N_4_OS, is nearly planar with the mean planes of the hy­droxy­benzyl and triazole rings inclined at an angle of only 3.2 (7)°. In the crystal, O—H⋯N hydrogen bonds between the hy­droxy group and the triazole ring in concert with weak N—H⋯S inter­molecular inter­actions between the triazole ring and thione group form chains along [-210] enclosing *R*_2_^2^(8) graph-set motifs. A weak intra­molecular C—H⋯S inter­action and inter­molecular π–π inter­actions [centroid–centroid distance = 3.5990 (15) Å] are also observed.

## Related literature   

For the chemistry of Schiff base compounds, see: Dubey & Vaid (1991 ▶); Yadav *et al.* (1994 ▶). For uses of Schiff bases in analytical applications and metal coordination, see: Galic *et al.* (2001 ▶); Wyrzykiewicz & Prukah (1998 ▶); Reddy & Lirgappa (1994 ▶). For the chemical and biological activity of Schiff base compounds, see: Barrera *et al.* (1985 ▶); Dornow *et al.* (1964 ▶); Malik *et al.* (2011 ▶); Thieme *et al.* (1973*a* ▶,*b* ▶); Wei & Bell (1982 ▶). For related structures see: Kant *et al.* (2012 ▶); Praveen *et al.* (2012 ▶); Kubicki *et al.* (2012 ▶); Jeyaseelan *et al.* (2012 ▶); Devarajegowda *et al.* (2012 ▶); Vinduvahini *et al.* (2011 ▶); Almutairi *et al.* (2012 ▶); Ding *et al.* (2009 ▶); Sarojini *et al.* (2007*a* ▶,*b* ▶). For standard bond lengths, see: Allen *et al.* (1987 ▶).
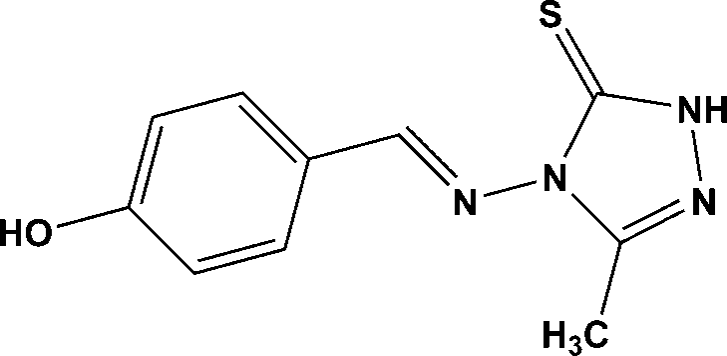


## Experimental   

### 

#### Crystal data  

C_10_H_10_N_4_OS
*M* *_r_* = 234.28
Triclinic, 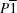

*a* = 5.7677 (5) Å
*b* = 7.7233 (8) Å
*c* = 12.7269 (12) Å
α = 84.104 (8)°
β = 77.719 (8)°
γ = 73.358 (9)°
*V* = 530.23 (9) Å^3^
*Z* = 2
Cu *K*α radiation
μ = 2.59 mm^−1^
*T* = 173 K
0.28 × 0.16 × 0.12 mm

#### Data collection  

Agilent Eos Gemini diffractometer
Absorption correction: multi-scan (*CrysAlis PRO* and *CrysAlis RED*; Agilent, 2012 ▶) *T*_min_ = 0.723, *T*_max_ = 1.000
3082 measured reflections
1987 independent reflections
1658 reflections with *I* > 2σ(*I*)
*R*_int_ = 0.030

#### Refinement  

*R*[*F* ^2^ > 2σ(*F* ^2^)] = 0.054
*wR*(*F* ^2^) = 0.151
*S* = 1.05
1987 reflections
147 parameters
H-atom parameters constrained
Δρ_max_ = 0.62 e Å^−3^
Δρ_min_ = −0.40 e Å^−3^

### 

Data collection: *CrysAlis PRO* (Agilent, 2012 ▶); cell refinement: *CrysAlis PRO*; data reduction: *CrysAlis RED* (Agilent, 2012 ▶); program(s) used to solve structure: *SUPERFLIP* (Palatinus *et al.*, 2012 ▶); program(s) used to refine structure: *SHELXL2012* (Sheldrick, 2008 ▶); molecular graphics: *OLEX2* (Dolomanov *et al.*, 2009 ▶); software used to prepare material for publication: *OLEX2*.

## Supplementary Material

Crystal structure: contains datablock(s) I. DOI: 10.1107/S1600536814012215/sj5405sup1.cif

Structure factors: contains datablock(s) I. DOI: 10.1107/S1600536814012215/sj5405Isup2.hkl

Supporting information file. DOI: 10.1107/S1600536814012215/sj5405Isup3.cml

CCDC reference: 1005409

Additional supporting information:  crystallographic information; 3D view; checkCIF report

## Figures and Tables

**Table 1:** Hydrogen-bond geometry (Å, °)

*D*—H⋯*A*	*D*—H	H⋯*A*	*D*⋯*A*	*D*—H⋯*A*
O1—H1⋯N3^i^	0.84	1.98	2.804 (3)	165
N4—H4⋯S1^ii^	0.88	2.46	3.324 (2)	166
C3—H3⋯S1	0.95	2.49	3.234 (3)	135

Symmetry codes: (i) 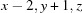; (ii) 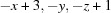.

**Fig. 1.**
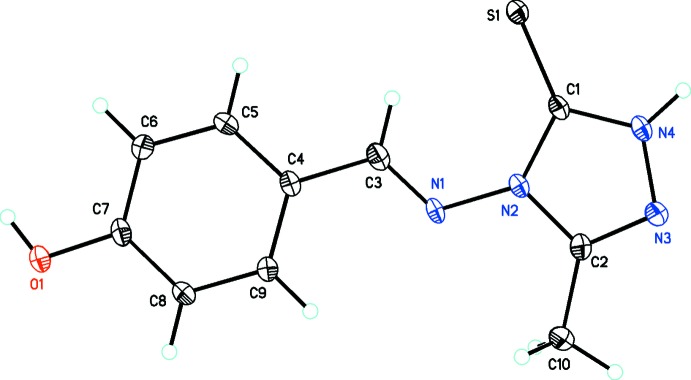
ORTEP drawing of (I), C10H10N4OS, showing the labeling scheme with 30% probability displacement ellipsoids.

**Fig. 2.**
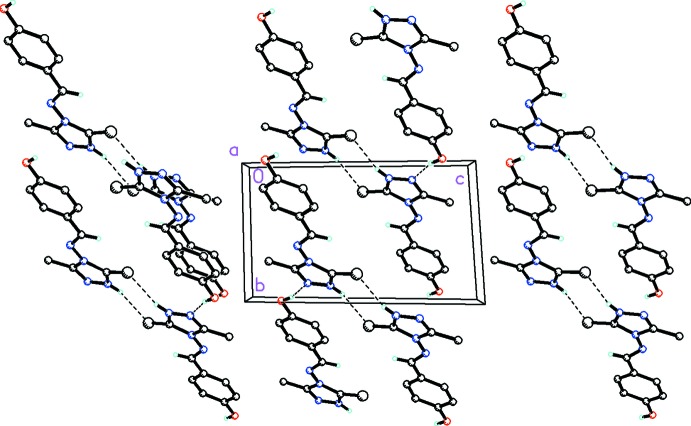
Molecular packing for (I) viewed along the b axis. Dashed lines indicate O—H···N hydogen bonds between the hydroxy group and triazole ring and weak S—H···S intermolecular interactions between the triazole ring and thione group forming infinite polymeric 1-dimensional chains along [210] and displaying R22(8) graph-set motifs. H atoms not involved in hydrogen bonding or weak intermolecular interactions have been removed for clarity.

**Fig. 3.**
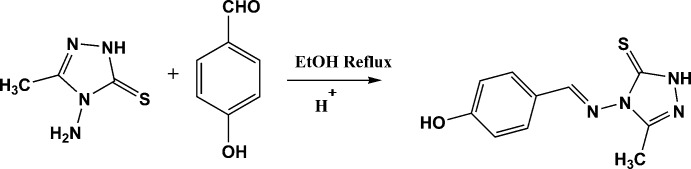
Reaction scheme.

## supplementary crystallographic information

### 1. Comment

During the last few decades, there has been a considerable interest in the chemistry of Schiff base compounds (Dubey & Vaid 1991; Yadav *et al.*, 1994). Schiff bases, containing different donor atoms, also find use in analytical applications and metal coordination (Galic *et al.*, 2001; Wyrzykiewicz & Prukah, 1998; Reddy & Lirgappa, 1994). Since many compounds containing sulfur and nitrogen atoms are antihypertensive (Wei & Bell, 1982), analgesic (Thieme *et al.*, 1973*a*,*b*), anti-inflammatory (Dornow *et al.*, 1964), sedative (Barrera *et al.*, 1985), or fungicidal (Malik *et al.*, 2011), synthesis of the corresponding heterocyclic compounds could be of interest from the viewpoint of chemical and biological activity. The crystal structures of some of the related Schiff bases viz: 3-ethyl-4-[(E)-(4-fluorobenzylidene)amino]-1H-1,2,4-triazole-5(4H)-thione (Jeyaseelan *et al.*, 2012); 4-[(E)-(4-fluorobenzylidene)amino]-3-methyl-1H-1,2,4-triazole-5(4H)-thione (Devarajegowda *et al.*, 2012); 3-[2-(2,6-dichloro-anilino)benzyl]-4-[(4-methoxybenzylidene)amino]-1H-1,2,4- triazole-5(4H)-thione (Vinduvahini *et al.*, 2011); 3-(adamantan-1-yl)-1-[(4-ethylpiperazin-1-yl)methyl]-4-[(E)-(4-hydroxy- benzylidene)amino]-1H-1,2,4-triazole-5(4H)-thione (Almutairi *et al.*, 2012); 4-{(2E)-2-[1-(4-Methoxyphenyl)ethylidene]hydrazinyl}-8-(trifluoromethyl) quinoline (Kubicki *et al.*, 2012); (E)-N'-(4-Methoxybenzylidene)-2-m-tolylacetohydrazide (Praveen *et al.*, 2012); (1Z)-1-[(2E)-3-(4-Bromophenyl)-1-(4-fluorophenyl)prop-2-en-1-ylidene]-2- (2,4-dinitrophenyl)hydrazine (Kant *et al.*, 2012); (E)-3-(2-ethoxyphenyl)-4-(2-fluorobenzylideneamino)-1H-1,2,4-triazole-5(4H)- thione (Ding *et al.*, 2009) have been reported. Crystal structures of some Schiff bases were also reported by our group (Sarojini *et al.*, 2007*a*,*b*). The present work describes the synthesis and crystal structure of the title compound, (I), C_10_H_10_N_4_OS.

In (I), the molecule is nearly planar with the mean planes of the hydroxybenzyl and triazole rings inclined at an angle of only 3.2 (7)°. (Fig. 1). Bond lengths are in normal ranges (Allen *et al.*, 1987). In the crystal, O—H···N hydrogen bonds between the hydroxy group and triazole ring in concert with weak N—H···S intermolecular interactions between the triazole ring and thione group form infinite polymeric 1-dimensional chains along [-2 1 0] displaying R_2_^2^(8) graph set motifs (Fig. 2). As the chains are extended, additional graph set motifs [R_4_^4^(28), R_4_^4^(30), R_4_^4^(32), R_6_^6^(50), R_6_^6^(52) & R_6_^6^(54)] are also formed. A weak C—H···S intramolecular interaction (Table 1) and weak π···π intermolecular interactions (Cg1–Cg2 = 3.5990 (15)Å, 1+x, y, z; (Cg1 and Cg2 are the centroids of the N2/C1/N3/N4/C2 and C4–C9 rings respectively) are also observed.

### 2. Experimental

To a suspension of 4-hydroxy benzaldehyde (1.22g, 0.01mol) in ethanol (15ml), 4-amino-5-methyl-2,4-dihydro-3H-1,2,4-triazole-3-thione (0.01mol, 1.3g) was added and heated to get a clear solution. To this a few drops of conc. H_2_SO_4_ was added as a catalyst and refluxed for 36 hr. on a water bath (Fig. 3). The precipitate formed was filtered and recrystallized from methanol to get the title compound, (I). Single crystals were grown from methanol by the slow evaporation method (m.p. 505–507 K).

### 3. Refinement

All of the H atoms were placed in their calculated positions and then refined using the riding model with Atom—H lengths of 0.95Å (CH), 0.98Å (CH_3_), 0.84Å (OH) or 0.88Å (NH). Isotropic displacement parameters for these atoms were set to 1.2 (CH, NH) or 1.5 (CH_3_, OH) times *U*_eq_ of the parent atom. Idealised Me and tetrahedral OH (O1(H1))were refined as rotating groups.

### Crystal data

**Table:**

C_10_H_10_N_4_OS	*Z* = 2
*M**_r_* = 234.28	*F*(000) = 244
Triclinic, *P*1	*D*_x_ = 1.467 Mg m^−^^3^
*a* = 5.7677 (5) Å	Cu *K*α radiation, λ = 1.54184 Å
*b* = 7.7233 (8) Å	Cell parameters from 1294 reflections
*c* = 12.7269 (12) Å	θ = 6.0–71.1°
α = 84.104 (8)°	µ = 2.59 mm^−^^1^
β = 77.719 (8)°	*T* = 173 K
γ = 73.358 (9)°	Prism, colourless
*V* = 530.23 (9) Å^3^	0.28 × 0.16 × 0.12 mm

### Data collection

**Table:**

Agilent Eos Gemini diffractometer	1987 independent reflections
Radiation source: Enhance (Cu) X-ray Source	1658 reflections with *I* > 2σ(*I*)
Graphite monochromator	*R*_int_ = 0.030
Detector resolution: 16.0416 pixels mm^-1^	θ_max_ = 71.3°, θ_min_ = 3.6°
ω scans	*h* = −6→7
Absorption correction: multi-scan (*CrysAlis PRO* and *CrysAlis RED*; Agilent, 2012)	*k* = −8→9
*T*_min_ = 0.723, *T*_max_ = 1.000	*l* = −11→15
3082 measured reflections	

### Refinement

**Table:**

Refinement on *F*^2^	Primary atom site location: structure-invariant direct methods
Least-squares matrix: full	Hydrogen site location: inferred from neighbouring sites
*R*[*F*^2^ > 2σ(*F*^2^)] = 0.054	H-atom parameters constrained
*wR*(*F*^2^) = 0.151	*w* = 1/[σ^2^(*F*_o_^2^) + (0.0895*P*)^2^ + 0.0331*P*] where *P* = (*F*_o_^2^ + 2*F*_c_^2^)/3
*S* = 1.05	(Δ/σ)_max_ = 0.001
1987 reflections	Δρ_max_ = 0.62 e Å^−^^3^
147 parameters	Δρ_min_ = −0.40 e Å^−^^3^
0 restraints	

### Special details

**Table:**

Geometry. All esds (except the esd in the dihedral angle between two l.s. planes) are estimated using the full covariance matrix. The cell esds are taken into account individually in the estimation of esds in distances, angles and torsion angles; correlations between esds in cell parameters are only used when they are defined by crystal symmetry. An approximate (isotropic) treatment of cell esds is used for estimating esds involving l.s. planes.

### Fractional atomic coordinates and isotropic or equivalent isotropic displacement parameters (Å^2^)

**Table:**

	*x*	*y*	*z*	*U*_iso_*/*U*_eq_	
S1	1.11567 (12)	0.19885 (9)	0.52135 (5)	0.0379 (3)	
O1	−0.1143 (3)	0.9783 (3)	0.86418 (16)	0.0377 (5)	
H1	−0.2048	1.0077	0.8180	0.057*	
N1	0.9248 (4)	0.4001 (3)	0.76560 (17)	0.0283 (5)	
N2	1.1501 (4)	0.2819 (3)	0.72431 (16)	0.0256 (4)	
N3	1.5264 (4)	0.1215 (3)	0.73740 (18)	0.0307 (5)	
N4	1.4752 (4)	0.1066 (3)	0.63900 (17)	0.0300 (5)	
H4	1.5835	0.0420	0.5881	0.036*	
C1	1.2477 (4)	0.1981 (3)	0.6267 (2)	0.0265 (5)	
C2	1.3255 (4)	0.2280 (3)	0.7878 (2)	0.0287 (5)	
C3	0.7774 (5)	0.4764 (3)	0.7032 (2)	0.0318 (6)	
H3	0.8179	0.4491	0.6293	0.038*	
C4	0.5459 (4)	0.6061 (3)	0.7454 (2)	0.0276 (5)	
C5	0.3644 (5)	0.6680 (4)	0.6829 (2)	0.0322 (6)	
H5	0.3944	0.6250	0.6126	0.039*	
C6	0.1427 (5)	0.7901 (3)	0.7209 (2)	0.0316 (6)	
H6	0.0210	0.8298	0.6773	0.038*	
C7	0.0976 (4)	0.8550 (3)	0.8236 (2)	0.0289 (5)	
C8	0.2773 (5)	0.7967 (3)	0.8869 (2)	0.0327 (6)	
H8	0.2482	0.8420	0.9566	0.039*	
C9	0.4976 (5)	0.6732 (3)	0.8483 (2)	0.0324 (6)	
H9	0.6186	0.6329	0.8923	0.039*	
C10	1.2826 (5)	0.2836 (4)	0.9004 (2)	0.0384 (6)	
H10A	1.1664	0.2240	0.9467	0.058*	
H10B	1.2139	0.4151	0.9029	0.058*	
H10C	1.4392	0.2481	0.9259	0.058*	

### Atomic displacement parameters (Å^2^)

**Table:**

	*U*^11^	*U*^22^	*U*^33^	*U*^12^	*U*^13^	*U*^23^
S1	0.0299 (4)	0.0437 (4)	0.0324 (4)	0.0105 (3)	−0.0096 (3)	−0.0190 (3)
O1	0.0277 (10)	0.0413 (11)	0.0346 (10)	0.0111 (8)	−0.0076 (8)	−0.0141 (8)
N1	0.0209 (10)	0.0271 (10)	0.0295 (11)	0.0057 (8)	−0.0011 (8)	−0.0113 (8)
N2	0.0212 (10)	0.0252 (9)	0.0255 (10)	0.0022 (8)	−0.0022 (8)	−0.0090 (8)
N3	0.0262 (11)	0.0329 (11)	0.0286 (11)	0.0026 (9)	−0.0061 (8)	−0.0104 (8)
N4	0.0229 (10)	0.0309 (10)	0.0295 (11)	0.0047 (8)	−0.0016 (8)	−0.0127 (8)
C1	0.0229 (11)	0.0252 (11)	0.0257 (11)	0.0025 (9)	−0.0016 (9)	−0.0085 (9)
C2	0.0223 (12)	0.0283 (12)	0.0314 (13)	0.0013 (9)	−0.0059 (10)	−0.0056 (10)
C3	0.0280 (13)	0.0294 (12)	0.0313 (13)	0.0013 (10)	−0.0011 (10)	−0.0077 (10)
C4	0.0224 (12)	0.0270 (11)	0.0280 (12)	0.0022 (9)	−0.0030 (9)	−0.0066 (9)
C5	0.0322 (13)	0.0355 (13)	0.0240 (12)	0.0016 (11)	−0.0048 (10)	−0.0121 (10)
C6	0.0265 (13)	0.0353 (13)	0.0291 (13)	0.0022 (10)	−0.0093 (10)	−0.0056 (10)
C7	0.0218 (12)	0.0258 (11)	0.0334 (13)	0.0021 (9)	−0.0024 (10)	−0.0063 (10)
C8	0.0293 (13)	0.0347 (13)	0.0300 (13)	0.0033 (11)	−0.0070 (10)	−0.0154 (11)
C9	0.0251 (13)	0.0350 (13)	0.0324 (13)	0.0060 (10)	−0.0099 (10)	−0.0118 (11)
C10	0.0342 (15)	0.0457 (15)	0.0285 (13)	0.0058 (12)	−0.0087 (11)	−0.0133 (12)

### Geometric parameters (Å, º)

**Table:**

S1—C1	1.675 (2)	C4—C5	1.396 (4)
O1—H1	0.8400	C4—C9	1.401 (4)
O1—C7	1.354 (3)	C5—H5	0.9500
N1—N2	1.388 (3)	C5—C6	1.378 (4)
N1—C3	1.267 (3)	C6—H6	0.9500
N2—C1	1.392 (3)	C6—C7	1.394 (4)
N2—C2	1.374 (3)	C7—C8	1.393 (4)
N3—N4	1.369 (3)	C8—H8	0.9500
N3—C2	1.295 (3)	C8—C9	1.379 (3)
N4—H4	0.8800	C9—H9	0.9500
N4—C1	1.334 (3)	C10—H10A	0.9800
C2—C10	1.488 (4)	C10—H10B	0.9800
C3—H3	0.9500	C10—H10C	0.9800
C3—C4	1.454 (3)		
			
C7—O1—H1	109.5	C4—C5—H5	119.3
C3—N1—N2	119.5 (2)	C6—C5—C4	121.4 (2)
N1—N2—C1	133.6 (2)	C6—C5—H5	119.3
C2—N2—N1	118.42 (19)	C5—C6—H6	120.1
C2—N2—C1	108.01 (19)	C5—C6—C7	119.7 (2)
C2—N3—N4	104.1 (2)	C7—C6—H6	120.1
N3—N4—H4	122.8	O1—C7—C6	122.3 (2)
C1—N4—N3	114.5 (2)	O1—C7—C8	117.8 (2)
C1—N4—H4	122.8	C8—C7—C6	119.8 (2)
N2—C1—S1	130.18 (18)	C7—C8—H8	120.1
N4—C1—S1	127.45 (18)	C9—C8—C7	119.9 (2)
N4—C1—N2	102.3 (2)	C9—C8—H8	120.1
N2—C2—C10	123.3 (2)	C4—C9—H9	119.5
N3—C2—N2	111.1 (2)	C8—C9—C4	121.1 (2)
N3—C2—C10	125.6 (2)	C8—C9—H9	119.5
N1—C3—H3	120.2	C2—C10—H10A	109.5
N1—C3—C4	119.6 (2)	C2—C10—H10B	109.5
C4—C3—H3	120.2	C2—C10—H10C	109.5
C5—C4—C3	120.1 (2)	H10A—C10—H10B	109.5
C5—C4—C9	118.0 (2)	H10A—C10—H10C	109.5
C9—C4—C3	121.9 (2)	H10B—C10—H10C	109.5
			
O1—C7—C8—C9	−179.1 (2)	C2—N2—C1—S1	175.0 (2)
N1—N2—C1—S1	−4.7 (4)	C2—N2—C1—N4	−2.0 (3)
N1—N2—C1—N4	178.3 (2)	C2—N3—N4—C1	−0.9 (3)
N1—N2—C2—N3	−178.6 (2)	C3—N1—N2—C1	−12.7 (4)
N1—N2—C2—C10	2.8 (4)	C3—N1—N2—C2	167.6 (2)
N1—C3—C4—C5	−169.2 (2)	C3—C4—C5—C6	179.6 (2)
N1—C3—C4—C9	11.1 (4)	C3—C4—C9—C8	179.7 (3)
N2—N1—C3—C4	−177.4 (2)	C4—C5—C6—C7	0.5 (4)
N3—N4—C1—S1	−175.24 (19)	C5—C4—C9—C8	0.0 (4)
N3—N4—C1—N2	1.9 (3)	C5—C6—C7—O1	178.4 (3)
N4—N3—C2—N2	−0.5 (3)	C5—C6—C7—C8	0.3 (4)
N4—N3—C2—C10	178.1 (3)	C6—C7—C8—C9	−0.9 (4)
C1—N2—C2—N3	1.6 (3)	C7—C8—C9—C4	0.7 (4)
C1—N2—C2—C10	−177.0 (2)	C9—C4—C5—C6	−0.6 (4)

### Hydrogen-bond geometry (Å, º)

**Table:**

*D*—H···*A*	*D*—H	H···*A*	*D*···*A*	*D*—H···*A*
O1—H1···N3^i^	0.84	1.98	2.804 (3)	165
N4—H4···S1^ii^	0.88	2.46	3.324 (2)	166
C3—H3···S1	0.95	2.49	3.234 (3)	135

Symmetry codes: (i) *x*−2, *y*+1, *z*; (ii) −*x*+3, −*y*, −*z*+1.
